# The COVID‐19 pandemic as a catalyst for medical education innovation: A learner’s perspective

**DOI:** 10.1096/fba.2020-00133

**Published:** 2021-03-18

**Authors:** David J. Savage

**Affiliations:** ^1^ Internal Medicine University of California San Diego La Jolla CA USA

**Keywords:** GME, telemedicine, UME, virtual interviews, virtual learning

## Abstract

The COVID‐19 pandemic has been transformative for healthcare and medical education. Physician trainees and the education system that serves them adapted quickly so that trainees could finish the academic year on time and advance to the next phase of training without compromising clinical competency or public safety. Systemic changes have had the most significant impact on telemedicine training, virtual learning, secure testing, and the interview process for residency and fellowship training positions. Trainees are now getting regular, supervised practice experience with telemedicine. Some secure testing is being done remotely, without jeopardizing examination test items or trainee assessment. Attending physicians are experimenting with novel ways to engage learners with video for virtual rounds to keep the rounding team safe. Finally, the interview process for medical school, residency, and fellowship programs, which has traditionally been an expensive and travel‐laden process, has been made completely virtual for the first time ever. These changes have disadvantages, including a lack of social connection, potential distraction when learning remotely, and limited contact with a potential training program when interviewing by video. This perspective paper, written by a senior internal medicine resident, details his firsthand experience with these changes during the pandemic. It also looks forward to how the current changes will likely change medical education permanently and for the better.

## 1 INTRODUCTION

I was in the second half of my second year of internal medicine residency training at the Cleveland Clinic when the COVID‐19 pandemic struck. Tuesday, March 10 was the morning that all conferences and in‐person meetings at our institution were canceled. Suddenly, all learning and a considerable portion of ambulatory clinic needed to be virtual. There were many technology tools available, but it took time for the leaders of our residency program to adapt them to our needs. Now, 1 year later, students and residents nationwide have firsthand experience with many innovative changes in the medical education and practice environment. The pandemic demonstrated how health educators, administrators, trainees, and regulators can be progressive and agile during a public health emergency. It has also paved the way for changes that would have otherwise taken years to flourish. Many changes will likely outlast the present pandemic and include robust telemedicine training, virtual didactics, school‐ or home‐based secure examinations, and virtual interviews.

## 2 TELEMEDICINE TRAINING

I was a member of the inaugural Clinician‐Educator Track (CET) at the Cleveland Clinic. In February 2020, our CET team ran a weeklong workshop to brainstorm and develop an ambitious telemedicine project. We created a curriculum for the internal medicine residency program for the upcoming academic year. At the time, we knew that telemedicine was a fast‐growing tool,^1^ and we wanted residents to learn how to use it well. Originally, we planned a cautious, incremental role out of our curriculum that would have taken years to fully implement.

One month later, the whole world changed because of the COVID‐19 pandemic. There was new urgency to convert patient appointments to be virtual for every person's safety. The United States Centers for Medicare & Medicaid Services (CMS), which had been resistant to broadly funding telemedicine care in urban centers in the past,^1^ changed Medicare rules quickly to meet this need.^2^ Our resident‐run primary care clinics adapted to this new reality, too. The residency program worked with the CET residents to expedite the rollout of our nascent telemedicine curriculum. Cleveland Clinic worked quickly with Epic Systems (Verona, WI) to implement a secure video platform that would work seamlessly within the electronic medical record (EMR) software. By the start of April 2020, all residents and ambulatory faculty had received a telemedicine orientation, and they were performing at least two video appointments each clinic, if not more. Trainees, who can sometimes be resistant to an abrupt curriculum change, were flexible and eager to learn this new skillset. This quickly shifted public opinion about telemedicine. Our patients reported satisfaction with seeing their doctors from the safety of home, and nationally, a recent patient sentiment survey showed that a majority of respondents now prefer virtual care for some medical services.^3^

In July 2020, I moved to the University of California San Diego (UC San Diego) to complete my third year of residency training. From day one in my new ambulatory clinic, I was seeing at least two patients per day by video or phone. Like Cleveland Clinic, UC San Diego’s EMR had been newly equipped with video software to make this possible. The medical students, residents, and faculty preceptors at UC San Diego were very adept at providing telehealth care, and like me, they had learned these skills in a very brief period of time. The opportunity to become proficient in practicing telemedicine within a structured GME training program has been one notable benefit of this pandemic.

Telemedicine is now pervasive in specialty care, too. In September 2020, I worked with two UC San Diego oncologists who ran completely virtual clinics. I connected from a primary care examination room on campus, and they worked from home. I saw patients first, discussed the treatment plan with the oncologist by phone, and then we saw the patient together by video. As a learner, this was very effective. Patients consistently said they preferred to see their oncologist this way because it saved considerable travel and wait time. This is a substantial benefit in oncology where patients might otherwise travel great distances for a second opinion and/or to gain access to clinical trials.^4^ In short, educational and regulatory changes that would have taken years to make telemedicine possible in primary care and subspecialty training programs were accelerated to a timespan of months nationwide because of the pandemic. As a result, the present generation of students, residents, and fellows will be considerably more prepared to provide tele‐healthcare. The transitions between real and virtual care will also become more seamless as patients come to expect a mixture of in‐person and virtual encounters.^5^

Telemedicine broadens the geographic reach of physicians, but it may still be out of reach of patients needing care the most, including the elderly, disabled, uninsured, and/or socioeconomically disadvantaged. As we teach and practice telemedicine, we should be mindful of the barriers that make this care inaccessible for patients and creative in the ways we adapt. In my experience, patients with limited access to video technology, for example, are often still reachable by a telephone call. As of April 2020, CMS provides some reimbursement for certain audio‐only telephone encounters,^6^ but this may not be permanent or universal. Regardless of funding, trainees should be taught and encouraged to use their full array of tools—be it a phone call, two‐way video, or a hybrid of in‐person/virtual care—to connect with patients and address their needs. At UC San Diego, I have experienced a remarkable example of such creativity by volunteering with the Student‐Run Free Clinic (SRFC). During the pandemic, the SRFC became virtual by necessity. Faculty and resident physicians precept care remotely, while medical students are the front line telemedicine clinicians. Interpreters are available by phone when needed, and a small interdisciplinary team is present on site at the clinic to help with coordination of volunteers, medication refills, food deliveries, and case management. This virtual clinic meets an urgent community need, while simultaneously allowing all trainees to better comprehend the many challenges faced by patients with limited resources and no health coverage. The virtual SRFC has also opened up service opportunities to a broader network of trainees like me. The industriousness of the UC San Diego SRFC is just one example of how telemedicine can broaden the reach of health systems to the community and also meaningfully engage trainees in the care of patients with limited resources.

### 2.1 Recommendations

- Telemedicine care will be a permanent component of United States healthcare in the future, and it is important for students, residents, and fellows to learn how to provide it in a supervised environment.
- We should urge national policymakers and insurance providers to continue funding telemedicine care even after the pandemic resolves.
- Older and economically disadvantaged patients still face considerable barriers to accessing telemedicine care including access to inexpensive video‐enabled devices and internet access. These care gaps must be addressed as telemedicine matures.

## 3 VIRTUAL LEARNING

Didactics and bedside rounds in training programs were also drastically changed as a result of the pandemic. My residency programs in Cleveland and San Diego had to quickly pivot to learning online using platforms like Zoom and Microsoft Teams. This came with the advantages of being able to attend the conference from anywhere and being able to invite expert discussants from other institutions. During a fall 2020 hematology‐oncology grand rounds at UC San Diego, for example, we were joined by an expert in veno‐occlusive disease from Johns Hopkins in Baltimore, MD. The subsequent discussion on thromboembolism prevention for patients with cancer was much richer because of his participation.

Virtual rounding has been another creative adaptation for both residents and medical students. On the Cleveland Clinic hepatology service in April 2020, our attending oversaw virtual rounds to keep the team safe. Residents would pre‐round and see patients one‐on‐one, but then we would video conference for morning team rounds. Our attending would join remotely from his office. He would provide feedback on our care plans, and lead deeper discussions about the best evidence guiding our management choices. He could show slides and papers real time from his library using screen sharing. We learned a lot from these rounds and soon it seemed normal. After virtual rounds, our attending would see the patients by himself at the nursing unit to ensure social distancing practices by not having the entire team in each patient's room. Later in the afternoon, we would meet again virtually to do structured learning based on the afflictions of our patients. Our attending would bring up notable examination findings from his bedside visits, sometimes with pictures collected with patient permission, to help improve our diagnostic skills for patients with liver disease. Medical students, who were also sidelined on clinical rotations due to COVID‐19 restrictions,^7^ participated in and learned from these virtual rounds, too. This model provided considerable educational value, optimized patient care time, and protected the safety of both trainees and patients.

Given the abundance of data that is available on the modern EMR, a considerable amount can be learned from patients by doing asynchronous, virtual rounding. Still, there is no substitute for periodic face‐to‐face bedside observation of the learner performing a physical examination or counseling patients and their families.^8^ Thus, for virtual rounds to be effective, learners and educators should be proactive about soliciting and providing actionable feedback. In the case of hepatology rounds, residents on our team requested the attending physician's presence for key family meetings. The attending, in turn, selected patients he wanted to see together with residents and students to teach important physical examination skills. Virtual rounds were an efficient way to learn and serve patients safely during the pandemic, but they required blending virtual and in‐person learning. With further refinement, this may persist in some form well after the pandemic ends.

### 3.1 Recommendations

- Virtual rounding and conferences can provide opportunities for residents and students to learn asynchronously. This can increase time for patient care, allow social distancing when needed, and permit expert speakers to join conferences from afar.
- Learners at all stages need to have supervised observations of their physical examination and patient care skills at the bedside so that they may receive actionable feedback. This can be one‐on‐one and with a subset of patients as an alternative to the traditional model where a large team visits the bedside of every patient.

## 4 REMOTE SECURE EXAMINATIONS

Secure examinations like the annual internal medicine In‐Training Examination (ITE), the United States Medical Licensing Examination (USMLE) Step 1 and 2 examinations, and the USMLE Step 2 Clinical Skills (CS) examination were also impacted by the pandemic. Social distancing measures increased the risk of taking secure examinations in tight quarters. Test sponsors took innovative steps as a result. The 2020 internal medicine ITE was administered remotely for some test takers, and could be proctored by video.^9^ The National Board of Medical Examiners (NBME) did not have enough Prometric testing seats to accommodate USMLE demand, so it partnered with medical schools to offer it on‐site and began to explore remote proctoring.^10^ The USMLE Step 2 CS examination, which was originally offered at only five testing centers nationally, was suspended May 2020 for 12–18 months^11^ and later suspended indefinitely in late January 2021.^12^ Weeks later, the osteopathic medicine equivalent examination was also indefinitely suspended.^13^ In the meantime, standardized clinical skills assessments with simulated patients have continued by using the school‐based cumulative objective structured clinical examination (OSCE), which is offered at most US medical schools.^14^ Each of these changes demonstrated that test sponsors, schools, and examinees were willing to make quick changes in order to continue necessary assessment programs to ensure the safety of testing candidates, proctoring staff, and ultimately, the public.

Several of these changes may last well beyond the pandemic. The success of remote ITE and USMLE examinations may provide strong evidence for continuing this trend in the future. The USMLE Step 1 and 2 examinations, which cost test takers $645 each in 2020–2021,^15^ may be more affordable and accessible if they can be proctored on‐site at a medical school or remotely at home with a video. The now‐canceled USMLE Step 2 CS examination will save $1300 for US‐based trainees and $1565 for international test‐takers based on the 2019–2020 examination fees.^16^, ^17^ In the future, school‐based OSCEs can be augmented to provide a standardized, summative clinical skills examination experience onsite, with the flexibility to evaluate many different patient care scenarios.^18^ This approach can also provide actionable, growth‐oriented performance feedback to each test taker, and help educators build remediation plans for learners when necessary. The disruption caused by COVID‐19 has changed the testing landscape drastically and for the better for physicians‐in‐training.

### 4.1 Recommendations

- Remotely administered secure examinations may decrease testing cost and increase convenience to test takers. These efforts should continue after the pandemic subsides.
- The permanent suspension of the USMLE Step 2 CS examination and its osteopathic equivalent will spare physicians‐in‐training a considerable testing expense.
- Medical school‐based OSCEs can fill the clinical skills assessment gap, and schools will now have flexibility to assess a broader range of clinical scenarios and provide actionable feedback to learners.

## 5 VIRTUAL INTERVIEWS FOR RESIDENCY AND FELLOWSHIP POSITIONS

In March 2020, most meetings and didactic learning for graduate medical education (GME) and undergraduate medical education (UME) went online. Interviews for medical school, residency programs, and fellowship programs soon followed suit. The American Association of Medical Colleges (AAMC) encouraged “medical school and teaching hospital faculty to conduct all interviews with potential students, residents, and faculty in a virtual setting—either by phone or through video conferencing.”^19^ Likewise, residency and fellowship programs nationwide made their 2020–2021 interviews virtual.^20^

When I interviewed for internal medicine residency programs in the fall of 2017, each interview required time away from school and considerable out‐of‐pocket cost for travel and housing. In 2020, by contrast, I interviewed for hematology‐oncology fellowships completely online. Prospective programs used several innovative approaches to showcase their facilities, unique training offerings, and faculty from afar. Videos provided virtual tours, testimonials from current fellows, and/or an overview from the program's leaders. Slide decks, websites, and online course modules featured faculty biographies, research opportunities, example rotation schedules, and housestaff benefits information. These resources were sent a week or more advance of the scheduled fellowship interview. In this way, the process became asynchronous so that the interview day itself frequently consisted of only interviews, question and answer sessions, and opportunities to meet current trainees and faculty.

Interviewing from home for fellowship saved substantial time and money. In some cases, it was even possible to interview in the morning and then report for clinical duty in the afternoon, or to interview with multiple programs in a single day. My cost of applying for fellowship was limited to my application fees alone, and I did not need to budget extra days away from residency training for travel. The virtual interview process appreciably lowered the cost for medical school applicants, too, and likely made the process more equitable for candidates of limited financial means.^21^ The biggest disadvantage cited by many applicants this year was that it is hard to imagine life in a new city or the culture at a prospective training institution without visiting in‐person. However, for candidates like me targeting a particular geographic area, this was less of a problem. Ideally, both applicants and programs will have positive experiences with virtual interviews so that the interview process for post‐graduate positions will remain virtual, at least in part, indefinitely.

### 5.1 Recommendations

- Remote interviews for residency and fellowship programs have many benefits including substantial savings of cost and time, and the opportunity to learn about a program's offerings in advance so that interview days are focused solely on interviewing, asking clarifying questions, and networking with current trainees.
- In the future, interviews should be a hybrid of in‐person and virtual with no prejudice towards interviewees who opt for a virtual interview day.

## 6 MENTORSHIP IN CLINICAL PRACTICE AND MEDICAL DECISION MAKING

It will always be important to learn a diagnostic schema or “illness script” for clinical presentations. There is no substitute for independent adult learning to internalize the diagnosis differential, work‐up process, diagnostic criteria, and treatment options for common clinical presentations. While much of this information can be looked up and memorized, putting it into practice in a time efficient, cost aware manner requires guidance of experienced physicians. Consequently, there still remains an essential place for hands‐on clinical decision‐making teaching. The process of forming a well‐reasoned assessment and plan is the critical expertise that physicians bring to patient care. Trainees rely on their attendings to teach them the fine points of building and prioritizing a broad differential diagnosis, and then determining when one test or treatment plan is preferable based on prior experience and the medical literature. We also learn communication skills like conveying bad news effectively, managing a challenging family dynamic, delivering complex critical care, and handling ethical dilemmas from those teachers. These skills are learned through practice, and refined by focused feedback. No amount of technology will replace teaching this skillset, but asynchronous clinical learning may offer new opportunities for focused one‐on‐one interactions between the supervising physician and trainee for professional growth.

## 7 LIMITATIONS AND DISADVANTAGES OF EDUCATION INNOVATIONS DURING THE COVID‐19 PANDEMIC

The most palpable disadvantage to socially distanced learning and interviews is that it can be both lonely and tiring. Human beings use a variety of social and environmental cues during in‐person interactions. These cues are oftentimes absent or difficult to discern during video meetings.^22^ This contributes in part to “Zoom fatigue,”^22^ a reference to the now ubiquitous video conference software, after hours online. I certainly felt this way after a full day of video interviews or virtual conference presentations. Afterward, I felt exhausted and eager for in‐person human contact. As a transplant to a new program for my senior year of residency training, I commiserated with interns who found forming community with their peers challenging in this socially distanced environment. In the future, conferences, and classroom courses should blend virtual and in‐person offerings to keep learners engaged, break up a marathon video learning session, and help foster peer interactions that are important for mental wellness.

Learner disengagement or distraction is another downside of virtual learning. It is very easy to turn off the video, mute the microphone, and multi‐task. For some students and physicians with clinical duties, distance learning in a clinical care space often means that pages and messages for urgent patient care needs compete for our attention.^23^ This diminishes learning and makes teaching challenging.^24^, ^25^ It is therefore important for learners to determinedly sideline distractions and for teachers to use multi‐modal strategies to make learning interactive and engage the audience. The use of interactive questions (ex. Poll Everywhere), shorter conference times (ex. 30 min instead of one hour), direct callouts to participants by name, and breakout rooms are effective strategies to promote engagement in my experience. Using them effectively, however, requires that the teachers adapt as well.^26^ Ultimately, we will all benefit from the best practices in remote teaching developed this year.

Medical students were stressed frequently by this year's pandemic education changes. Some institutions accustomed to teaching anatomy by cadaveric dissection transitioned to virtual anatomy didactics for pre‐clinical students.^27^ Scheduled time for students on some clinical clerkships was decreased or staggered to minimize COVID‐19 exposure.^7^ This reduced the amount of patient contact and learning experiences available, even despite the use of virtual rounding previously described. Students nearing graduation were discouraged or prohibited from participating in fourth year away rotations because of the pandemic.^28^ Such rotations, while costly, are historically invaluable to being competitive for residency programs in certain fields.^29^ Finally, USMLE site cancelations led to considerable testing delays, provoking additional anxiety in the preparation for this high stakes examination series.^30^ In several cases, students had to extend their study period by months or start clinical clerkships without taking the Step 1 examination as they had previously planned. Such impacts were unavoidable, but the resulting adaptations—virtual sub‐internships,^31^, ^32^ disruptive changes in the selection process for residency program trainees,^33^ and partnerships between the NBME and medical schools for on‐site USMLE administration^10^—provide hints of how to make elements of UME less burdensome to student trainees after the pandemic.

Finally, as of this writing, we are in the midst of a “third wave” of rising COVID‐19 cases and It is taking a toll on healthcare providers, medical educators, and physician learners nationwide as we strain to serve our communities. There are undoubtedly many ways in which COVID‐19 has impacted UME and GME training for the better and worse that are not described here. However, I expect that many more medical education developments will be shared by others as we take stock of the breakthroughs, appreciated more thoroughly in hindsight, after this unprecedentedly bleak year.

## 8 CONCLUSION

The worldwide COVID‐19 pandemic that began in early 2020 created a challenging public health crisis for the world's health and medical education systems. It also created many new opportunities to advance undergraduate and graduate medical education using technology tools at a rapid pace. As a learner, I was the direct beneficiary of these changes, and I hope the future will be a hybrid of each of these advances. Learning telemedicine skills in clinic is invaluable, some classroom and ward learning is well suited to being online, licensing examination cost and time burdens can be reduced by remote administration, and virtual interviews for training positions are both time‐ and cost‐efficient. If used judiciously, these advances move professional training forward with numerous upsides. These adaptations are still in flux, but this trainee is confident and optimistic that the breakthroughs this year will leave an indelible positive mark on undergraduate and graduate medical education.

## CONFLICT OF INTEREST

DJS is the resident member of the AMA’s Council on Medical Education.

## AUTHOR CONTRIBUTIONS

DJS wrote and edited this manuscript.
